# Cell death-based treatments of melanoma:conventional treatments and new therapeutic strategies

**DOI:** 10.1038/s41419-017-0059-7

**Published:** 2018-01-25

**Authors:** Gianfranco Mattia, Rossella Puglisi, Barbara Ascione, Walter Malorni, Alessandra Carè, Paola Matarrese

**Affiliations:** Oncology Unit, Center for Gender-Specific Medicine, Istituto Superiore di Sanità Italian National Institute of Health, Viale Regina Elena 299, 00161 Rome, Italy

## Abstract

The incidence of malignant melanoma has continued to rise during the past decades. However, in the last few years, treatment protocols have significantly been improved thanks to a better understanding of the key oncogenes and signaling pathways involved in its pathogenesis and progression. Anticancer therapy would either kill tumor cells by triggering apoptosis or permanently arrest them in the G1 phase of the cell cycle. Unfortunately, melanoma is often refractory to commonly used anticancer drugs. More recently, however, some new anticancer strategies have been developed that are “external” to cancer cells, for example stimulating the immune system’s response or inhibiting angiogenesis. In fact, the increasing knowledge of melanoma pathogenetic mechanisms, in particular the discovery of genetic mutations activating specific oncogenes, stimulated the development of molecularly targeted therapies, a form of treatment in which a drug (chemical or biological) is developed with the goal of exclusively destroying cancer cells by interfering with specific molecules that drive growth and spreading of the tumor. Again, after the initial exciting results associated with targeted therapy, tumor resistance and/or relapse of the melanoma lesion have been observed. Hence, very recently, new therapeutic strategies based on the modulation of the immune system function have been developed. Since cancer cells are known to be capable of evading immune-mediated surveillance, i.e., to block the immune system cell activity, a series of molecular strategies, including monoclonal antibodies, have been developed in order to “release the brakes” on the immune system igniting immune reactivation and hindering metastatic melanoma cell growth. In this review we analyze the various biological strategies underlying conventional chemotherapy as well as the most recently developed targeted therapies and immunotherapies, pointing at the molecular mechanisms of cell injury and death engaged by the different classes of therapeutic agents.

## Facts

- Molecularly targeted therapy induces cell death of melanoma cells.
- Immunotherapy has demonstrated dramatic efficacy for several cancers including melanoma.
- There is a gender disparity in terms of response to therapy.

## Open questions

- To assess effectiveness and sustainability of immune-checkpoint inhibitors.
- To better characterize the tumor cell death and resistance mechanisms, e.g., by autophagy, induced by immunotherapy.
- To discover further mutated genes of interest for molecularly targeted therapy.
- Why the response to therapy of men and women is different?

## Introduction

Melanoma is the most aggressive skin cancer, originating from cutaneous, mucosal, and uveal melanocytes. Cutaneous melanoma arises from melanocytes and represents the most aggressive form of skin cancer. According to epidemiological data, 132,000 new cases of melanoma and 50,000 melanoma-related deaths are diagnosed worldwide each year^1^. Cumulative epidemiologic data from Europe and United States indicate a continuous and dramatic increase in incidence during the last decades (new cases per year: 13.2 per 100,000 subjects in Europe and 21.6 in US in 2012)^2^. Excluding familiar forms, cutaneous melanoma development is mainly affected by ultraviolet radiations. Others risk factors are multiple nevi, environmental exposure to toxic agents and immunosuppression.

As for other forms of cancer, melanoma progression depends upon a series of increasing survival-oriented molecular alterations resulting in the development of cancer cell clones selected for their ability to survive in an extremely unfavorable microenvironment and capable of overwhelm the lack of nutrients. Indeed, these cells can deceive host’s immune response, survive hypoxia, oxidative stress and induction of apoptosis, finally developing a remarkable propensity for metastatic spreading^3^, the most life-threatening event in melanoma patients.

During the last few years, treatment of melanoma in advanced phases has shown some improvement by the introduction of new therapeutic approaches, including target and immunological therapies, thus opening a new era for treating this aggressive form of cancer^4^.

## Conventional therapy: cytotoxic drugs

For long time, surgical resection of early tumors represented the sole therapeutic option and only later chemotherapy was introduced in the treatment of melanoma. Unfortunately, metastatic melanoma is often refractory to commonly used anticancer drugs^5^. The understanding of the mechanisms underlying this chemoresistance could improve clinical outcome and impact melanoma patient’s management in a cost-effective manner.

Resistance to cancer therapy, either intrinsic, due to cell clone selection, or acquired, due to the activation of alternative survival pathways, is a multifactorial process related not only to neoplasia subtype, tumor genotype and heterogeneity, but also to own patient’s features^6,7^. In fact, drugs are differently processed and metabolized in patients, possibly modifying both effectiveness and toxicity of treatments. These differences can be due to genetic and epigenetic backgrounds including sex-associated metabolic disparity. Moreover, although chemotherapy kills most cancer cells, it is thought to save tumor stem cells. These cells, representing the most drug-resistant population of the tumor, can trigger an important mechanism of resistance. It is then fundamental that anticancer strategies could target this cell population^6,7^.

There are several factors that can lead to drug resistance, such as disrupted apoptobsis machinery, overactive pro-survival signaling pathways, increased expression of the therapeutic target, activation of alternative compensatory pathways, a high degree of molecular heterogeneity, and upregulation of drug transporters^8^. Drug resistance has frequently been associated with genetic mutations and/or with abnormal expression of apoptosis-related molecules, such as FLIP, Bcl-2, Bcl-XL, MCL-1, p53, APAF-1, Bax, Fas, FADD, and caspases^9^.

Until a few years ago, it was believed that efficient anticancer regimens would either kill tumor cells, by engaging the apoptotic machinery, or permanently arrest them in the G1 phase of the cell cycle. More recently, it was observed that some anticancer agents can induce other forms of cell death, such as programmed necrosis or mitotic catastrophe-engaged apoptosis^10^. This aspect may be particularly interesting since: (i) necrosis could prove helpful in removing those cancer cells that have developed resistance to apoptosis, and (ii) cancer cells are particularly susceptible to the induction of mitotic catastrophe thanks to their genomic instability^11^. In fact, an entire class of anticancer agents, such as taxanes and vinca alkaloids, triggers mitotic catastrophe by binding to tubulin and disrupting the mitotic spindle^12^.

Nonetheless, since most, if not all, cancer cells exhibit or acquire increased resistance against pro-apoptotic agents, the future of anticancer therapy also relies on the exploitation of non- and pre-apoptotic signaling cascades. For instance, as mentioned above, another intensively studied programmed cell death pathway of interest in the field of oncology is called necroptosis, a process regulated via the RIPK1/RIPK3/MLKL activation pathway^13,14^. Of notice, this pathway is often deregulated in tumor cells, including melanoma cells in which RIPK3 expression is lacking^15,16^. Conventional pro-apoptotic agents, including TNF-related apoptosis-inducing ligand (TRAIL), the inhibitors of apoptosis protein inhibitors (IAP), Bcl-2 and several anticancer drugs can induce necroptosis, when apoptosis is blocked^17^. For example 5-Fluorouracil (5-FU) induces RIP1/MLKL-dependent necroptosis in caspase-3-deficient cancer cells^18^, whereas cisplatin (CDDP) caused RIP3-dependent necroptosis in apoptosis-resistant cancer cells through necrosome formation and autocrine TNF-α signaling^19^. Interestingly, necroptosis is often accompanied by autophagy, which may be responsible for suppression of apoptosis and bias toward necroptosis.

As concerns autophagy, although its cytocidal potential remains rather controversial, recent studies dealing with agents capable of modulating autophagic process appear as promising^20^. In fact, preclinical studies have implicated a potential tumor suppressive function of autophagy in the initiation of tumor formation, but a protective role favoring tumor cell survival once the tumor has already formed^21^. Several recent publications reported autophagy as a protective mechanism against chemotherapy-induced cell death in melanoma^22–26^. However, the role of autophagy in promoting melanoma cell death induced by different cytotoxic compounds has also been described^27–29^. For instance, Lakhter and colleagues showed that chloroquine, raising the lysosomal pH, inhibited autophagy^30^ promoting apoptosis in vitro and inhibiting melanoma tumor growth in vivo^31^. Very recently, it was also reported that nutrient deprivation could significantly enhance sensitivity of melanoma cells to chemotherapy-induced death. Although autophagy was known to be activated by nutrient deprivation, these authors found that, at least in their in vitro cell model, autophagy did not contribute to enhance sensitivity of melanoma cells to cisplatin^32^. However, the role of autophagy in melanoma is far from being clarified and further in vivo experiments appear as mandatory.

## Chemotherapy of melanoma

A major form of chemotherapy for melanoma includes pro-apoptotic drugs (e.g., cisplatin, 5-fluorouracil). Cisplatin is a platinum derivative cis-diamminedichloroplatinum(II) (CDDP) that, when activated, passively diffuse into the cytosol^33^. Used in the clinical management of different types of cancer, including melanoma, this drug generates irreparable DNA damage, inducing either a permanent proliferative arrest, i.e., cellular senescence, or activation of the mitochondrial pathway of apoptosis. Indeed, more recent studies suggest that the cytostatic and cytotoxic activities of CDDP involve not only nuclear, but also cytoplasmic mechanisms^34^ eventually promoting a persistent oxidative stress, which may result in direct cytotoxic effects or indirectly provoking DNA damage^35^.

5-fluorouracil (5-FU), together with capecitabine and others, belongs to the chemotherapeutic agents targeting the enzyme thymidylate synthase (TS) and the thymidine monophosphate. The inhibition of TS results in deficiency of thymidylate, imbalance in cellular nucleotide pools and impairment of DNA replication and repair, thus inducing cell-cycle arrest and DNA damage^36,37^. Unfortunately, in melanoma, intratumoral thymidylate synthase overexpression is highly induced in response to treatment with 5-FU and other thymidylate synthase inhibitors discouraging their use^38^.

Few years ago, alkylating agents with cytostatic activity were introduced as the only standardized therapeutic option in clinical management of melanoma^39^. Temozolomide (TMZ) and dacarbazine (DTIC) were preferentially used, but the overall success was very limited in metastatic melanoma^40^. DTIC is the only FDA-approved chemotherapy for melanoma, but it has not been shown to improve progression-free or overall survival (OS) in randomized clinical trials^41^. Resistance to alkylating agents, associated with increased expression of the DNA repair protein O6-alkylguanine DNA alkyltransferase (MGMT), represents in fact a fairly frequent occurrence in melanoma^42^.

As in general cancer cells proliferate faster and with less error-correcting than healthy cells, they result more sensitive to DNA damage. Nonetheless, alkylating antineoplastic agents as those mentioned above are also toxic to normal cells (cytotoxic) that divide frequently, such as those of mucosa, bone marrow, testicles and ovaries, causing a number of side effects, including loss of fertility. In addition, most of the alkylating agents are carcinogenic per se and can be associated with the development of secondary malignancies.

## Targeted therapy: inhibitors of the BRAF pathway

Targeted therapy works differently from standard chemotherapy, which basically attacks any rapidly dividing cells^43^. Indeed, the goal of targeted therapies is to exclusively destroy cancer cells. These agents (chemical or biological) are designed to interfere with those molecules specifically driving growth and spreading of the tumor. A targeted therapy approach represents a personalized treatment, as each patient receives drugs based on the unique genetic profile, or subtype, of its tumor.

The mitogen-activated protein kinase (MAPK) signaling pathway is an important mediator of cell proliferation and differentiation in melanoma. MAPKs are serine-threonine kinases that mediate intracellular signaling associated with a variety of cellular activities, including cell proliferation, differentiation, survival, death, and transformation. In particular, extracellular signal-regulated kinase (ERK), which belongs to the MAPK family, plays a role in several key steps of tumor development. For instance, ERK-dependent phosphorylation of proteins, such as myosin light chain kinase, calpain, focal adhesion kinase, and paxillin^44^, promotes cancer cell migration and increases the expression of matrix metalloproteinases by promoting degradation of extracellular matrix proteins and consequent tumor invasion^45^. ERK1/2 also regulate the activities and levels of Bcl-2 family proteins thus promoting cancer cells survival^46^. In 2002, the discovery that 40–60% of cutaneous melanomas harbor activating mutations in the serine/threonine kinase gene BRAF^47^ made possible the development of specific drugs, which were tested in a series of clinical trials that ultimately led the Food and Drug Administration (FDA) to approve the BRAF inhibitor vemurafenib (Zelboraf, Genentech/Roche, South San Francisco, CA), the first drug to come out of fragment-based drug discovery^48^. BRAF is a member of the RAF family, along with ARAF and CRAF (also called RAF1) proteins, which are involved in directing cell growth. Mutations have been described at a number of sites in the BRAF gene, with about 80% resulting in the substitution of glutamic acid (E) for valine (V) in codon 600, the BRAF V600E mutation^47^. Other common BRAF mutations were found at the same V600 codon (V600K, about 16% of mutations and V600D/R, 3% of all mutations in melanoma), with slightly higher rates in melanomas arising in older patients^49^. All of these V600 mutations result in a mutant form of the BRAF protein that is constitutively active. Actually, the first BRAF inhibitor tested in patients with melanoma, sorafenib, showed little efficacy^50^ either alone or combined with other conventional chemotherapeutic agents^51^. However, at present, the beneficial effects of BRAF inhibitors in melanoma patients bearing BRAF V600 mutations are well established. Nonetheless, the main issue remains the development of drug resistance, which is responsible for disease relapse within months after treatment. In most cases BRAF resistant melanomas bear additional mutations reactivating MAPK pathway, e.g., MEK1 mutations, and BRAF or KRAS amplification^52^. The observed frequent co-activation of MEK in BRAF resistant tumors led to the development of combination therapies with BRAF inhibitor plus MEK inhibitors (e.g., trametinib), which improve survival, but are unable to prevent disease relapse^53^.

Although combination therapies that simultaneously block multiple pathways may display improved efficacy by making more difficult for tumor cells to escape destruction, they are often associated with relevant side effects^54^. Unexpectedly, the association of BRAF and MEK inhibitors was less toxic than BRAF monotherapy. This is consistent with results of primary trials and reflects the BRAF-inhibitor–induced paradoxical activation of the MAP kinase pathway, which causes skin-related toxic effects, including secondary cutaneous malignancy^55,56^. In addition, it is important to underscore that a new generation of BRAF inhibitors (i.e., PLX8394 and PLX7904), able to circumvent the paradoxical activation of MAPK pathways, is under development.

Other common mutations in melanoma (15–20%) are in the NRAS gene. Interestingly, melanoma with NRAS mutations virtually never presents BRAF mutations^57^. This feature could make these tumors potentially eligible for a targeted therapy. Unfortunately, mutations in NRAS lead to up regulation of heterogeneous effector pathways, thus making drug development more difficult.

Acral lentiginous and mucosal melanomas harbor, more frequently than others, KIT mutations (8–17%)^58^. Some of these melanomas are sensitive to treatment with imatinib mesylate (Gleevec, Novartis, Basel, Switzerland), a multikinase inhibitor targeting Abl and KIT, as well as with platelet-derived growth factor receptor inhibitors, such as sorafenib^59^. However, KIT-directed therapy has been disappointing compared with selective BRAF inhibitors.

In sum, as mentioned above, after initial enthusiastic results, when used as single agents, targeted therapies were unable to show statistically improved OS and progression-free survival (PFS), and tumor resistance and recrudescence of disease were often observed^60,61^.

Drug-induced resistance was observed either after long-term in vitro treatment of tumor cell lines or in in vivo models^62^. In melanoma cell lines treated with sub-lethal concentrations of vemurafenib, it was associated with upregulation of stem cell markers and downregulation of differentiation markers^63^. The involvement of the melanoma transcription factor MITF, providing resistance to MAPK-pathway inhibitors through various mechanisms including survival signals, was also reported^64–66^. Accordingly, enhanced MITF expression was linked with innate resistance, and MITF amplification and/or increased expression were found in some advanced melanomas^67^. In addition, the acquisition of cell resistance cannot be linked to a clear genetic cause, but rather to epigenetic changes. Drugs can induce an epigenetic reprogramming, converting the transient transcriptional state to a stably resistant one^68^.

Very interestingly, it was observed that the acquired tumor resistance to BRAF plus MEK inhibition could be reversible, and that patients with BRAFV600-mutant melanoma can respond when rechallenged with dabrafenib plus trametinib. This represents the first prospective trial to show that rechallenge with any targeted treatment can reinduce tumor responses after a treatment interruption^69^. Finally, it was also recently observed that the HIV1-protease inhibitor nelfinavir, was able to sensitize BRAF and NRAS mutant melanoma cells to MAPK-pathway inhibitors. Nelfinavir was also found effective in BRAF/NRAS/PTEN mutant tumors^70^. This feature represents a typical case of the so-called drug repositioning.

**Fig. 1:**
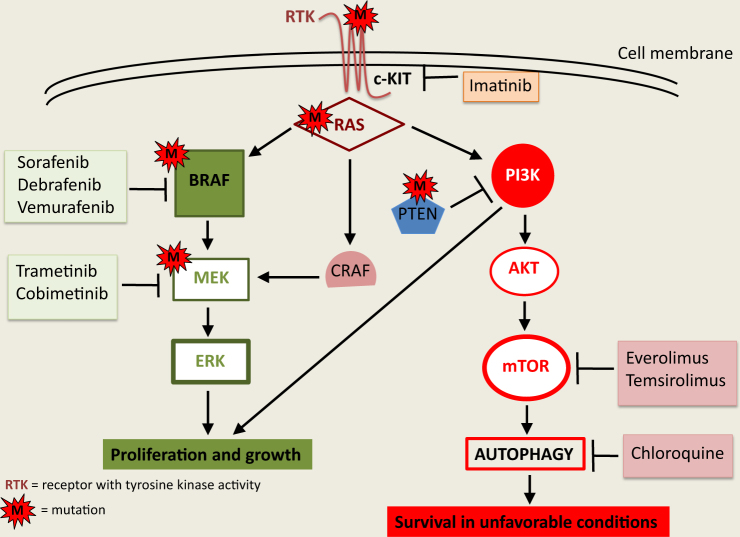
Functional mechanisms of targeted therapy Binding of ligands to receptors with tyrosine kinase activity (e.g., c-KIT) promotes the activation of downstream signaling pathways, including RAS, CRAF, MEK, ERK, PI3K, and AKT as key molecules. Inhibition by Imatinib or by different BRAF and MEK inhibitors represents clinically relevant strategies

The mechanisms of targeted therapy are schematically represented in Fig. 1.

## Immunotherapy

Approximately 40 years of studies have dissected the molecular mechanisms connecting tumor, microenvironment, and different types of immune cells, thus encouraging the development of different forms of immunotherapy. Recently, with the advent of therapeutic immune checkpoint inhibitors, immunotherapy against some key molecules is emerging as the elective option for melanoma treatment^71^.

### Immuno-mediated mechanisms of tumor cell death

Although it is well known that cancer cells develop strategies to evade immune-mediated killing, the discovery of immune checkpoint blockade made the immune reactivation a more conceivable antitumor action. The principal effectors of the enhanced antitumor immune response are fully activated CD8^+^ Cytotoxic T lymphocytes (CTLs) and Natural Killer (NK) cells whose action can cause tumor cell death^72,73^. Two basic mechanisms exist for killing target cells: lytic and apoptotic cell death. In the lytic cell death, specific tumor associated antigens (TAAs) are recognized by activated CD8^+^ CTLs that release lytic granules containing perforin and granzyme B, capable to lysate target cells. Beyond the antigens, CTLs can recognize the FAS Ligand (FAS L) death receptor on target cells. FAS:FAS L interaction induces transmission of the death signal to tumor cells bringing to apoptosis^74^. In parallel, activated NKs are able to recognize tumor cells independently from Major Histocompatibility Complex (MHC) as normally do the CTLs. This occurs when tumors escape the killing action of CTLs avoiding the presentation of TAAs together with MHC^75^. NK cells are capable to release granules that induce apoptotic cell death when recognize TAAs on tumor cells through a mechanism of antibody dependent cytotoxicity^76,77^.

### Melanoma immuno-escape mechanisms

At its initial stage, melanoma is considered one of the most immunogenic type of cancer as revealed by: (i) occasional remission and presence of lymphocytic infiltrates both in primary and metastatic areas; (ii) impossibility to find the original primary tumor after its dissemination perhaps in view of the antitumor action of the immune system; (iii) isolation of tumor T lymphocytes recognizing specific melanoma antigens; (iv) melanoma capability to respond to immunotherapy^71^. Unfortunately, with malignant evolution, melanoma cells escape immunosurveillance by manipulation of local and systemic microenvironment, eventually destroying innate and adaptive immune responses. The dysfunctional state of T cells has been termed ‘exhaustion’, on the basis of similarities to chronic infections^78^. This phenomenon depends on different mechanisms originated by infiltrating innate immune cells and tumor cells through the action of cytokines, chemokines and nutrients released in the tumor microenvironment. In particular, T-reg lymphocytes promote immunosuppression impairing activation, survival and expansion of antitumor CTLs through the production of transforming growth factor-β (TGF-β) and interleukin-10 (IL-10), considered immunosuppressive cytokines^79^. T cell dysfunction is also obtained by immature antigen presenting cells^80^ and by Myeloid-derived suppressor cells^81^. Furthermore, melanoma cells undergoing Epithelial-Mesenchymal transition (EMT) escape from T cells killing by EMT-dependent down-regulation of tumor antigen expression^82^.

In healthy subjects, T-cell activation is strongly regulated by the expression of cytotoxic T-lymphocyte antigen-4 (CTLA-4), one of the most important co-receptor inhibitors controlling immune response. This molecule competes with CD28 on antigen presenting cells (APC), causing inhibitory signaling of T cell activation by blocking Interleukin 2 (IL-2) expression and cell division. This mechanism, important for immune tolerance and adaptive immune resistance, is responsible for a fatal restriction on initiating an efficacious immune response against tumor cells. A second critical aspect in the tumor–immune system interface comprises the interaction of the activated effector T cells with target cells, which principally takes place in the inflamed microenvironment where primed lymphocytes recognize specific antigens^83^.

Differently from CTLA-4, Programmed Death-1 (PD-1, also named CD279) checkpoint attenuates the action of stimulated effector T lymphocytes to avoid host tissue damage. In presence of a tumor, the PD-1 signal leads to a diminished antitumor response and activated T cell anergy^84^. Functionally, T cells express PD-1 that, interacting with its ligands PD-L1 or PD-L2 (B7-H1/CD274 or B7-DC/CD273) on tumor cells, induces a tolerance state of tumor infiltrating T lymphocytes that are less capable of carrying out antitumor immunity. This condition has been associated with poorer patients’ outcome^85^.

**Fig. 2:**
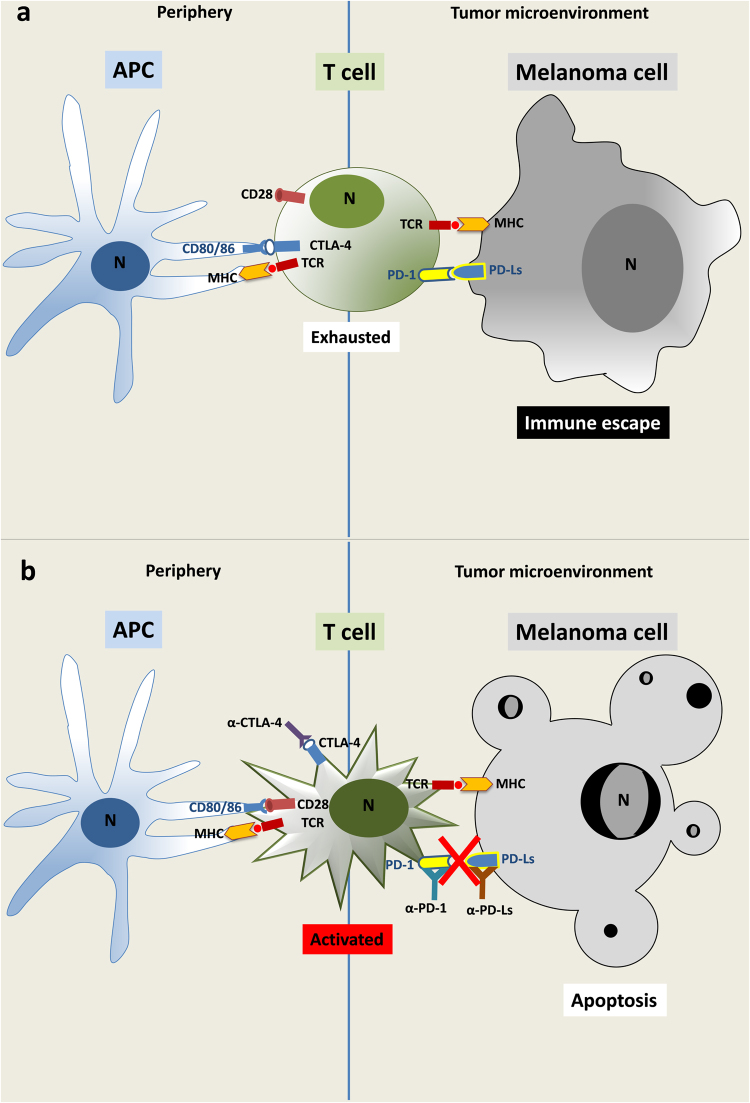
Immune checkpoint modulation of the T cell activity **a** APCs, loaded with antigenic peptides for presentation to the TCR by MHC, are unable to activate T cells in peripheral lymphoid organs through CD80/86:CD28 co-stimulatory signals. This inhibition is due to CTLA-4 sequestration of CD80/86 molecules (left). In tumor microenvironment, PD-L1/L2 expressed by melanoma cells link the co-inhibitory PD-1 molecule on activated T cells limiting their effects against tumor cells. This process can eventually lead to T cell exhaustion and immune escape of tumor cells (right). **b** T cell activation is obtained either in peripheral lymphoid organs (left) or in the tumor microenvironment (right) by anti-CTLA-4 or anti-PD-1 and anti-PD-L1 or -L2 antibodies, respectively. The abrogation of each immune checkpoint pathway by interruption of CTLA-4:CD80/86 or PD-1:PD-L1/L2 binding restores the immune response against melanoma cells

In the past 20 years a lot of melanoma antigens have been associated with tumor infiltrating lymphocytes (TIL), either proteins of melanocyte differentiation (gp100, tyrosinase and Melan-A) or aberrantly expressed melanoma associated genes (MAGEs)^86–91^. In this expectantly state, different immunotherapeutic approaches were developed starting from cytokine treatment alone or in combination with classical chemotherapy, peptide-protein-tumor cell vaccines or adoptive cell therapy with lymphocyte activated killer (LAK) cells and melanoma specific T cell clones. All these approaches had as main goal the reactivation of the killing functions of the immune system against tumor cells. Except for high dose of IL-2, the majority of these trials did not provide a real therapeutic advantage^71^. Thus, in view of the growing comprehension on CTLA-4 and PD-1 inhibitory checkpoints during tumor immune response, a new interest for immunotherapy of tumors has led to the development of co-inhibitory antibodies to re-engage the immune system, impeding its exhausted state and favoring the reactivation of their lytic and/or pro-apoptotic functions against tumor cells^92–94^. Fig. 2 shows a schematic representation of the immune checkpoint functional modulations.

## Anti-CTLA-4 immunotherapy

The rationale of using anti-CTLA-4 antibody in the treatment of melanoma is based on the general concept that tumor immunotherapy may eventually promote tumor growth as consequence of incorrect and prolonged immune response^95,96^. Thus, the antibody blocking CTLA-4 inhibitory checkpoint avoids immunosuppressive state of lymphocytes, strengthening their antitumor action. The key role of this molecule in immune response was evidenced for the first time in CTLA-4-deficient mice that, after antigen exposure, developed a severe and lethal lymphoproliferative disorder due to persistent T cell proliferation and activation in peripheral tissues^97^. In addition in B16 melanoma mouse models, the use of anti-CTLA-4 monoclonal antibody after vaccination with irradiated GM-CSF-transduced tumor cells was sufficient to induce tumor eradication, although the treatment produced a severe autoimmune disease with depigmentation or vitiligo CD8^+^ lymphocytes-dependent^98^.

Ipilimumab (Yervoy®), the best studied anti-CTLA-4 monoclonal antibody, was evaluated in different clinical trials in various tumors. Based on two phase III randomized trials demonstrating improvement on median OS over control arms, the therapeutic use of ipilimumab was approved as first (US) or second line (European Union) treatment for management of unresectable or metastatic stage IV melanoma^93^. In a prospective study on patients at stage IV of melanoma, a 5% statistically significant improvement of OS at 3-years was obtained in patients receiving ipilimumab in combination with dacarbazine compared to dacarbazine alone or placebo groups^40^. In a recent study in patients with advanced melanoma, Ascierto and coauthors demonstrated that ipilimumab significantly increased the overall survival^99^. Another monoclonal antibody, named tremelimumab (ticilimumab, CP-675,206), showed evidence of tumor regression in a phase I trial, although with more severe immune-related side effects compared with ipilimumab^100^. A second study on stage IV melanoma patients with tremelimumab as first line therapy in comparison with dacarbazine, demonstrated not statistically significant differences in OS, although patients with objective response to tremelimumab had longer duration (35.8 months) compared with patients responding to dacarbazine (13.7 months)^101^.

## Anti-PD-1 immunotherapy

The second important checkpoint with strategic relevance for antitumor therapy is the reactivation of effector T lymphocytes by PD-1:PD-L1 pathway inhibition. This pathway maintains T cell tolerance to preserve peripheral tissues from autoimmunity. In vitro and in vivo preclinical studies suggested the possibility of blocking PD-1:PD-Ls interactions for relieving the immunosuppressive effects and enhancing the cytotoxic activity of antitumor T cells^102^. PD-1 is a transmembrane protein with immunoreceptor tyrosine-based inhibitory signaling, identified as an apoptosis-associated molecule^103^. It is expressed on cell surface of CD4, CD8, B lymphocytes, NK cells, monocytes and dendritic cells, following activation^104^. In 2001, a second ligand for PD-1, named PD-L2, was discovered^105^. PD-L1 is constitutively expressed on different hematopoietic cells as well as on fibroblasts, endothelial cells, mesenchymal cells, neurons and keratinocytes^106,107^. Differently, PD-L2 is expressed on activated DCs, macrophages, mast cells and activated B cells. Notably, PD-Ls are also expressed on several tumor cells favoring their association with activated T lymphocytes and the consequent anergic effect^108^. The PD-1:PD-L1 interaction is active only in presence of T or B cell antigen receptor crosslink. This interaction prevents PI3K/Akt signaling and MAPK/ERK pathway activation with the net result of lymphocytic functional exhaustion^109,110^.

Different antibodies have been developed to block PD-1 checkpoint. The response was significant in different tumors, including melanoma, with better clinical benefit and minor toxicity compared to anti-CTLA-4 therapy. Nivolumab (Opdivo®) was the first antibody developed against PD-1 and utilized in clinical trials for treatment of melanoma, renal cell carcinoma (RCC) and non-small cell lung cancer (NSCLC)^111^. Nivolumab treatment in phase Ib demonstrated highly specific action, durable tumor remission and long term safety in 32% of patients with advanced melanoma^112^. Two phase III studies on melanoma patients were conducted with nivolumab compared with dacarbazine, either on patients with wild type or mutated BRAF, the latter unresponsive to ipilimumab. On naive patients with metastatic melanoma, results obtained have shown a higher response rate with nivolumab vs dacarbazine (40% vs. 14%)^113^. Accordingly, complete or partial responses were more evident in the nivolumab group than in the chemotherapy group, irrespective of BRAF status or previous anti-CTLA-4 benefits. Importantly, the efficacy of the treatment with nivolumab was proportional to the expression level of PD-L1^114^. In fact, in 2014, the FDA approved nivolumab for treatment of patients with advanced and unresponsive melanoma. A recent clinical trial combining ipilimumab and nivolumab resulted in an impressive increase of PFS compared to ipilimumab given alone (11.5 months vs. 2.9)^115^. Particularly relevant was the improvement in term of PFS and OS associated with pembrolizumab (Keytruda®), a second anti-PD-1 antibody utilized for treatment of advanced melanoma compared to anti-CTLA-4 therapy^113,116^. In the same year pembrolizumab was also approved by the FDA for treatment of advanced melanoma in patients previously treated with ipilimumab or BRAF inhibitors in BRAF V600 mutation positive patients.

## Immunotherapy alternative targets

Despite the impressive impact of CTLA4- and PD1:PDL1-targeted cancer immunotherapy, a significant proportion of patients, including those with melanoma, failed to respond. Consequently, the focus has shifted to alternative inhibitory targets and suppressive mechanisms within the tumor microenvironment. LAG3 is a CD4 homolog that binds MHC class II molecules on macrophages and DCs. LAG3 is expressed in all classes of activated lymphocytes, including NK cells, where attenuates expansion and level of activation. LAG3 expression induces T regulatory function to disadvantage of CD8 + effector T cells allowing tumor cells to escape immune response^117^. Interestingly blockage of LAG3 activity or LAG3 knockout mice reverse the unresponsive state of T cells without signs of autoimmunity^118^.

T-cell membrane protein 3 (TIM3) is expressed by different types of immune cells and its ligands are galectin-9 and high mobility group box 1 proteins^119,120^. TIM3 is expressed on melanoma cells and frequently co-expressed with PD-1 on CD8 + T cells. As for LAG3, simultaneous targeting of TIM3 and PD-1 increased immunotherapeutic response^121^.

Further new strategies have been developed as alternative methods to obtain properly activated T lymphocytes. For instance, tumor specific antigen receptors, derived from tumor specific T cell clones, are genetically engineered in T lymphocytes, forming a chimeric antigen receptor (CAR) that allows the generation of T cells targeting tumor^122^. CARs combine antigen-specificity with T cell activation signal in a single fusion molecule that is retrovirally and stably expressed by T cells. Generally, molecules of the TCR signaling machinery are used, as CD3ζ or CD28, to permit satisfactory T cell activation able to recognize and kill tumor cells^123^. For melanoma, proteins utilized in TCR fusion constructs for TILs activation are MART-1, Ny-eso-1 and MAGE-A3^124–126^.

## Epigenetic modifications

Cutaneous melanoma is also influenced by epigenetic events affecting key cellular pathways co-responsible of disease development and progression. MicroRNAs (miRNAs) are small non coding RNAs (21–25 bp) that post-transcriptionally regulate gene expression. They possess oncogenic or tumor suppressor activity in various tumors, including melanoma, where their epigenetic regulation has been associated with progression and metastatization. Although often limited to cell lines, miRNA profiling demonstrated extensive modifications of their expression in melanoma compared to their “normal” counterpart, i.e., melanocytes, or in the different phases of progression^127^. MiR-15b and miR-155 have clearly been associated with apoptotic pathways, although with opposite roles. MiR-15b is up-regulated in advanced melanoma and its downregulation associated with reduced proliferation and increased apoptosis^128^. On the contrary, miR-155 is down-regulated in different cell lines with respect to melanocytes and its ectopic re-expression significantly inhibited cell growth^129^.

Although attractive, a therapeutic use of single miRNAs to restore (mimic) or abrogate (antagomiR) their expressions has not been fully developed, taking in mind the high number of genes that each single miRNA can regulate possibly loosing action specificity.

Genes specifically involved in cell cycle, differentiation, apoptosis and immune recognition can be modulated by DNA methylation and histone acetylation status. Pharmacological inhibition of DNA methyltransferase or histone deacetylases by demethylating and acetylating agents (i.e., 5-Aza-deoxycytidine (5-AZA-dC) and the hydroxamic acid Tricostatin A (TSA)) might re-establishes the expression of aberrantly silenced genes, restoring normal pathway functions. One of the clearer examples of DNA methylation, affecting the apoptotic program in melanoma, is the silencing of CDKN2A locus, encoding for the tumor suppressor genes, p16^INK4A^ and p14^ARF^. These genes are respectively methylated in 27 and 57% of metastatic melanomas, prevalently as a result of deletion of one allele and hypermethylation of the remaining one. As a consequence, melanoma cells escape from growth arrest and apoptosis generated by pRB and p53 pathways^130^. Although demethylation of these and other tumor suppressor genes with pro-apoptotic function (RASSF1A and TRAIL, for example) was able to restore cell death pathways, clinical studies on this matter in melanoma are still lacking^131^.

## A look at gender differences

Although with some variations across the world, significant differences have been noted between men and women in melanoma incidence^132–134^. In addition, although melanoma can arise everywhere in the human body, in women it is more common on the extremities and in men on trunk, head and neck^135^. A further medical conundrum is represented by the role played by patient’s sex in the prognosis, progression and survival. In fact, the survival advantage, even 45%^136^, for female patients persists after adjustment for several other prognostic indicators such as age, Breslow thickness, ulceration and localization of the primary tumor^137^. Hence, the better prognosis for women appears as not related to a more aggressive primary tumor at diagnosis, but it seems to be associated with lower propensity to metastasize. In this regard, published data seem to suggest that biological differences between the two sexes in disease-host interaction could be related to a complex framework of agents, including estrogen and androgen levels, estrogen receptors expression, reactive oxygen species generation, matrix metalloproteinase-2 (MMP-2) expression, apoptosis susceptibility, skin physiology and immune system function (higher in females). Despite all these data, sex-tailored therapeutic strategies are still lacking^138^. More recently, however, Gupta and co-workers, analyzing several whole exome sequencing datasets for cutaneous melanoma, determined that genomic differences actually exist between males and females. In fact, they found that male tumors harbor a higher mutation burden than female ones. In particular, they observed a statistically significant greater burden of missense mutations among men, even after adjusting for age at diagnosis, primary tumor site, stage at diagnosis, site of sequenced tumor, history of neoadjuvant treatment, and BRAF and NRAS mutation status. Interestingly, this gender-associated differential mutation burden, although evaluated in 19 different cancers, was found as specific for cutaneous melanoma^139^. The link between mutation burden and immune response may explain, at least in part, the female survival advantage observed clinically. In fact, the work by Youlden and collaborators reported that female patients with melanoma had a statistically significantly higher frequency of tumor-associated, antigen-specific CD4 + T-cells than their male counterparts^140^. This issue could be of great relevance in the era of immunotherapy and immune check point inhibitors in cancer treatment^141,142^.

## Perspectives

**Fig. 3:**
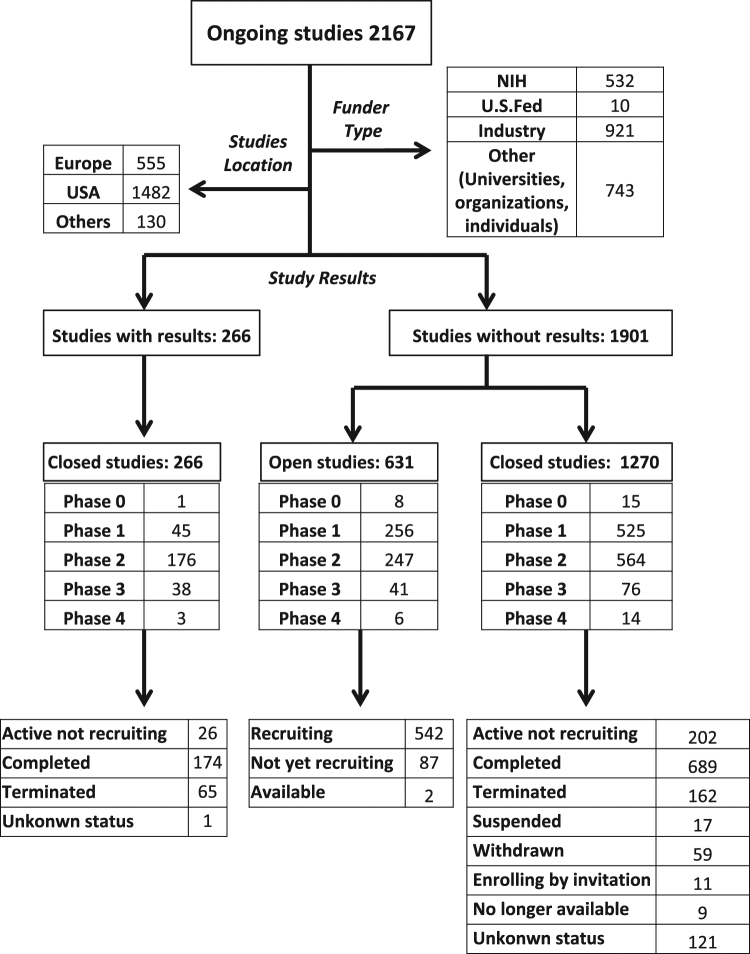
Worldwide clinical trials for melanoma treatment (updated June 2017) The flowchart illustrates the worldwide clinical trials considering different funder types, phase and status of the studies (from clinicaltrial.gov)

The future of melanoma therapy is either to develop new drugs or to improve the use of those readily available. The goal of each therapeutic schedule should overcome the disappointing results associated with the unsuitable molecular signatures connected to the problematic classification of this heterogeneous tumor. A better patient stratification would make possible to assess the best suited drug combinations, particularly for treatment of stage III or IV metastatic unresectable melanoma or for improving the median recurrence-free survival of stage III resected melanoma. Combined therapies have often demonstrated improvement of OS and/or PFS using either immune checkpoint inhibitors or target therapy drugs. At present, only 11 therapeutic choices against melanoma have been approved for clinical use, including BRAF and MEK inhibitors and therapeutic immune checkpoint inhibitors as well as IL-2 or Interferon alpha (Table 1). Although showing promising results, these options represent very exiguous weapons to win metastatic melanoma due to its high heterogeneity, problematic patient stratification and high genetic mutational rate. At present, more than 2000 trials are ongoing, and, among 1527 closed studies, > 250 have results. The majority of these studies are in phase I or II and only 23 in phase IV (Fig. 3). It is evident that the high costs of this enormous scientific work unavoidable fall back into society of every single country. One example is the treatment with checkpoint inhibitors that, in immediate near future, could be a real option for long term cure of advanced tumors, including melanomas. Nivolumab plus ipilimumab was shown to yield a median PFS of 11.5 months for metastatic melanoma cure, a disease stadium retained up to few years ago fatal and incurable^143^. Now, the problem is that compared to kinase inhibitors, immunological therapies have increased their costs, which appear unsustainable also for rich society (about 250,000 vs. 100,000 Euro/patient for year of therapy, respectively). It is therefore obvious the necessity that health system institutions and pharmaceutical industry discuss to license new promising drug with more accessible prices thus assuring long term cancer therapy for everyone and minimize disparity in health care^143^.

**Table 1:** Therapeutic drugs for treatment of melanoma

Drugs	Efficacy	Clinical indications	Approval by FDA
Dacarbazine (DTIC-Dome)	Alkylating agent ("antineoplastic" or "cytotoxic")	Advanced metastatic melanoma	1975
Interferon alfa-2b (Intron A®)	Adjuvant therapy for patients with high-risk of melanoma recurrence	Resected melanoma (stage IIb, IIc and III)	1995
Proleukin (Aldesleukin®)	Improved immune response with some cases of CR	Advanced metastatic melanoma	1998
Vemurafenib (Zelboraf®)	First drug to come out of fragment-based drug discovery	Unresectable melanoma with BRAF V600E	2011
	Improved OS and PFS versus conventional therapy	Only approved for BRAF mutant melanoma	for research studies only
Ipilimumab	MoAb anti CTLA-4	Unresectable advanced metastatic melanoma	2011
(Yervoy®)	Adjuvant Therapy		
Dabrafenib (Tafinlar®)	Improved OS and PFS versus conventional therapy	Unresectable melanomas with BRAF V600.	2013
		Not indicated for wild-type BRAF	
Trametinib (Mekinist®) MEK inhibitor	Improved OS and PFS *versus* conventional therapy	Unresectable or metastatic melanoma with BRAF V600E or V600K mutations.	2013
		Not indicated for the treatment of patients who have received a prior BRAF inhibitor therapy	
Dabrafenib (Tafinlar®) +Trametinib (Mekinist®)	Randomized trials in progress *versus* Dabrafenib monotherapyImprove survival but are unable to prevent disease relapse	Unresectable or metastatic melanomas with BRAF V600E or V600K mutation	Accelerated approval in 2013
Nivolumab (Opdivo®)	Anti PD-1 immune checkpoint inhibitor Significant increase of OS and PFS versus conventional chemotherapy	Advanced metastatic melanoma including Ipilimumab treatment refractory ones	2014
Pembrolizumab	Anti PD-1 immune checkpoint inhibitor	Unresectable Stage III and Stage IV melanoma	2014
(Keytruda®)	Significant increase of PFS versus Ipilimumab treatment		
Vemurafenib (Zelboraf®)+Cobimetinib (Cotellic®)	Improved PF and OS versus Vemurafenib monotherapy	BRAF V600 mutant melanoma	2015
Nivolumab (Opdivo®) + Ipilimumab (Yervoy®)	Combined treatment more effective than each drug alone. Increased PFS and OS	Unresectable Stage III and Stage IV melanoma PD-L1 negative melanoma	2015
